# AucPR: An AUC-based approach using penalized regression for disease prediction with high-dimensional omics data

**DOI:** 10.1186/1471-2164-15-S10-S1

**Published:** 2014-12-12

**Authors:** Wenbao Yu, Taesung Park

**Affiliations:** 1 Department of statistics, Seoul National University, Shilim-dong, Kwanak-gu 151-742, Seoul, Korea; 2 Interdisciplinary Program in Bioinformatics, Seoul National University, Seoul, Korea

**Keywords:** AUC, high-dimensional data, penalized regression, ROC curve

## Abstract

**Motivation:**

It is common to get an optimal combination of markers for disease classification and prediction when multiple markers are available. Many approaches based on the area under the receiver operating characteristic curve (AUC) have been proposed. Existing works based on AUC in a high-dimensional context depend mainly on a non-parametric, smooth approximation of AUC, with no work using a parametric AUC-based approach, for high-dimensional data.

**Results:**

We propose an AUC-based approach using penalized regression (AucPR), which is a parametric method used for obtaining a linear combination for maximizing the AUC. To obtain the AUC maximizer in a high-dimensional context, we transform a classical parametric AUC maximizer, which is used in a low-dimensional context, into a regression framework and thus, apply the penalization regression approach directly. Two kinds of penalization, lasso and elastic net, are considered. The parametric approach can avoid some of the difficulties of a conventional non-parametric AUC-based approach, such as the lack of an appropriate concave objective function and a prudent choice of the smoothing parameter. We apply the proposed AucPR for gene selection and classification using four real microarray and synthetic data. Through numerical studies, AucPR is shown to perform better than the penalized logistic regression and the nonparametric AUC-based method, in the sense of AUC and sensitivity for a given specificity, particularly when there are many correlated genes.

**Conclusion:**

We propose a powerful parametric and easily-implementable linear classifier AucPR, for gene selection and disease prediction for high-dimensional data. AucPR is recommended for its good prediction performance. Beside gene expression microarray data, AucPR can be applied to other types of high-dimensional omics data, such as miRNA and protein data.

## Background

Nowadays, it is easy and common to measure thousands of markers simultaneously through high-throughput technologies, for example, the microarray study. A disease is usually related to several markers and the combination of multiple makers for classifying a subject into different statuses of a specific disease is widely studied. The performance of a combination of markers is frequently measured by indices related to the Receiver Operating Characteristic (ROC) curve: sensitivity, specificity, or the area under the ROC curve (AUC). Sensitivity (specificity) is defined as the probability of success in classifying a diseased (non-diseased) individual accurately. By varying the decision rules (thresholds), different sensitivities and specificities are obtained. The ROC curve plots all possible sensitivities against 1-specificities and expresses the trade-off between sensitivity and specificity visually. AUC is the most popular summary index for the curve; it has been shown to be the probability that the score of a randomly chosen diseased individual exceeds that of a randomly chosen non-diseased subject [1].

Therefore, it is natural to construct a combination of markers in order to maximize the ROC-based metrics. A number of combinations based on ROC indices have been suggested by [2-9]. Among these, [3] and [5] developed distribution-free methods to achieve the best linear combination for maximizing the smoothed AUC for high-dimensional situations. They developed algorithms based on optimizing a sigmoid approximation of AUC. The sigmoid approximation of AUC relies on a smoothing parameter, which should be carefully chosen, though there are no theoretical guidelines for choosing this parameter. The rule of thumb for the choice of the smoothing parameter may reduce the power of the method. Moreover, the sigmoid approximation of AUC is not a concave function and multiple local maxima may exist. The global maximum is not guaranteed to be attained through commonly used numeric algorithms. For example, the performance of the linear combination decided by [5] is very poor for microarray data [9]. To avoid the difficulties of maximizing a non-parametric approximation of AUC, we can use a parametric method. To our knowledge, there is no published parametric method for maximizing the AUC under a high-dimensional context. This paper tries to fill this gap.

We suggest an AUC-based approach using penalized regression (AucPR), based on a classical parametric linear combination derived by [2] in a low-dimensional context. The problem is then transformed into a linear regression framework, and the existing software for solving linear regression with penalization can be used directly, which facilitates the implementation of the proposed method. There are many penalty functions available, for example, the elastic net criterion [10], which is a mixture of penalties of *L*_1 _and *L*_2 _norms of the linear coefficients. The lasso penalty [11] is a special form of elastic net. Both the lasso and the elastic net have been widely used for marker selection and disease classification for high-dimensional data [3,5,9,10,12,13]. In this work, we maximize AUC through elastic net or lasso penalty. We compare the proposed AucPR to a logistic regression with elastic net or lasso penalty and the AUC-based non-parametric method proposed by [3], through four microarray data sets and synthetic data. The performance is gauged on the AUC and sensitivity given specificity equals to 0.95 on testing samples. AucPR achieves better prediction performance.

## Methods

### AucPR: An AUC-based approach using penalized regression

Suppose non-diseased samples {***X_i_***; 1 ≤ *i *≤ *m*} and diseased samples {***Y****_j_*; 1 ≤ *j *≤ *n*} are independent and identically distributed (i.i.d.) from multivariate normal distributions *N *(***µ****_x_*, **Σ***_x_*) and *N *(***µ****_y _*, **Σ***_y _*), respectively, where ***µ****_x _*and ***µ****_y _*are *p*-dimension mean vectors, and **Σ***_x _*and **Σ***_y _*are*p × p *covariance matrices.

Under the multivariate normal distribution assumption, [2] showed that, among all possible linear combination of markers, without a positive constant multiplier, the combination with the coefficient vector

(1) $$\beta ={\left(\underset{x}{\sum }+\underset{y}{\sum }\right)}^{-1}\left(\mu_{y}-\mu_{x}\right)$$

is optimum for maximizing the AUC. Furthermore, they also proved that if **Σ***_x _*is proportional to **Σ***_y_*, ***β ***is uniformly optimum, that is, it achieves the highest ROC curve among all linear combinations for all possible values of specificity.

Although this approach has been widely used in disease classification [14-17], it cannot be applied directly to high-dimensional problems, where the number of markers (p) are larger than the number of observations in the sample. Penalized regression methods such as lasso [11] and elastic net [10], are effective tools for variable selection in high-dimensional problems. We thus try to restate our problem in a regression framework.

Note that from Equation (1), ***µ****_y _− **µ**_x _*= (**Σ**_*y *_+ **Σ**_*x*_)***β ***holds. Instead of solving this equation, we suggest approximating *β *by solving the following linear regression problem:

(2) $$\mu_{y}-\mu_{x}=\left(\sum_{y}+\sum_{x}\right)\beta +\in ,\;\in \sim N\left(0,\sigma^{2}I\right),$$

where ***I ***is a *p × p *identity matrix. By this transformation, we can avoid calculating the inverse of a large covariance matrix in (1), which is intractable due to lack of samples.

We then propose using a regularized linear regression method to obtain ***β***. Let **Σ **= **Σ***_y _*+ **Σ***_x _*= ((*σ_ij_*)), 1 ≤ *i, j *≤ *p*, and ***µ ***= ***µ****_y _− **µ**_x _*= (*µ*_1_*, . . . , µ_p_*)ʹ. Then, using the elastic net, we have

(3) $$\beta =\text{arg}\;\underset{\beta \in R^{p}}{\text{min}}\overset{p}{\underset{j=1}{\sum }}{(\mu_{i}-\overset{p}{\underset{j=1}{\sum }}\sigma_{ij}\beta_{j}\text{)}}^{2}+\lambda \left(\alpha \overset{p}{\underset{i=1}{\sum }}|\beta_{i}|+\frac{1-\alpha }{2}\overset{p}{\underset{i=1}{\sum }}\beta_{i}^{2}\right),$$

where λ is a parameter controlling the strength of the penalty and *α *is a mixing parameter that determines the relative strength of the *L*_1 _norm to the *L*_2 _norm, with 0 ≤ *α *≤ 1. When *α *= 1, the elastic net reduces to lasso. The elastic net encourages a group of highly correlated markers to enter the model together, while lasso is quite parsimonious in selecting correlated markers. Under some conditions, both penalties were shown to have consistency in model selection [18,19], or in other words, the selected model includes the true model with a high probability.

In practice, we replace the covariance matrices with the sample covariance matrices, and the mean vectors with the sample mean vectors. Formally,

$${\hat{\mu }}_{x}=\frac{1}{m}\overset{m}{\underset{i=1}{\sum }}X_{i},\;{\hat{\mu }}_{y}=\frac{1}{n}\overset{n}{\underset{i=1}{\sum }}Y_{i},$$

$${\hat{\sum }}_{x}=\frac{1}{m-1}\overset{m}{\underset{i=1}{\sum }}\left(X_{i}-{\hat{\mu }}_{x}\right){\left(X_{i}-{\hat{\mu }}_{x}\right)}^{\prime },$$

$${\hat{\sum }}_{y}=\frac{1}{n-1}\overset{n}{\underset{i=1}{\sum }}\left(Y_{i}-{\hat{\mu }}_{y}\right){\left(Y_{i}-{\hat{\mu }}_{y}\right)}^{\prime },$$

and

$$\hat{\mu }={\hat{\mu }}_{y}-{\hat{\mu }}_{x},\;\hat{\sum }={\hat{\sum }}_{y}+{\hat{\sum }}_{x}.$$

The idea of the proposed AucPR is similar to a procedure proposed by [20] for the sparse linear discriminant analysis (LDA), where they restrict *L*_∞ _error and obtain the combination by linear programming. When Σ*_x _*and Σ*_y _*are proportional to each other, **Σ **^− 1^***µ ***is proportional to the coefficient vector of LDA. In this sense, AucPR also provides a solution for sparse LDA.

There are several computationally efficient algorithms to implement penalized linear regression for high-dimensional data, for example, program *lars *by [21] and *glmnet *by [22]. In this paper, we use *glmnet *to solve Equation (3), since it is more efficient than *lars *[22].

*Remark 1: *We use sample mean vectors and sample covariance matrices, which are quite sensitive to the outlier observations. Therefore, intuitively, it may lead to the proposed method being inefficient under a general mean and a covariance structure without any restriction, especially when the sample sizes are small. However, AucPR can be powerful for some structures of **Σ **and ***µ***, for example, when **Σ **or ***µ ***are sparse, which is common in high-dimensional data. We illustrate this with numerical studies in the Result and discussion Section.

### Choice of tuning parameter

The tuning parameter λ controls the trade-off between data fitting and model complexity. Given a larger λ, fewer markers are selected and the data may not be well fitted, while for a smaller λ, a larger number of markers are chosen and overfitting may occur. We tune λ in our numeric studies by a three-fold cross-validation (CV) method. Note that when the sample sizes are large, we can use a *K*-fold CV with *K >*3.

For the *K*-fold CV, we randomly divide the samples into *K *subsets of equal size. We select λ that maximizes the following CV score:

(4) $$CV_{\lambda }=\overset{k}{\underset{i=1}{\sum }}{\hat{AUC}}_{\lambda }^{\left(i\right)}\left({\hat{\beta }}_{\lambda }^{\left(-i\right)}\right),$$

where ${\hat{\beta }}_{\lambda }^{\left(-i\right)}$ is the coefficient vector estimated without the samples in the *i*-th fold, and ${\hat{AUC}}_{\lambda }^{\left(i\right)}$ is the empirical AUC estimator with the data in the *i *-th fold, for a given λ, *i *= 1*, ... , K*. The empirical AUC estimator for a given ***β ***is defined as $\sum \sum I\left(\beta^{\prime }\left(Y_{i}-X_{j}\right)>0\right)/nm$, with *I *(·) being the indicator function.

For the elastic net, *α *is fixed at 0.5 in this investigation. We note that although *α *can be tuned in the same fashion as λ, a simple, fixed *α *still captures the characteristics of the elastic net and is widely used in the literature as well [13,23].

Another practical issue about tuning the parameter λ is how to provide the candidates of λ for CV, as it has not been specified clearly in the literature. We propose finding the range of λ using the whole data, and then generating a fixed number of candidates within that range such that they are evenly distributed in the log scale. Denoting the range of candidates for λ as [λ*_l_*, λ*_u_*], where λ*_l _*corresponds to the most complex model (for example, 100 markers are selected) while λ*_u _*corresponds to the least complex model (for example, 1 marker is chosen). It is easy to use the bisection method [24] to fix λ = λ*_k_*, such that there are exactly *k *non-zero coefficients (*k *= 1*,..., p*). To do this, we first have an initial guess at the value of λ. Let *r*(λ) be the number of non-zero coefficients of the tuning parameter λ. If *r*(λ) *= k*, we are done. If *r*(λ) *< k*, we let λ = λ*/*2, continuing this until *r*(*λ*) ≥ *k*. Once we have an interval [*λ*_1_*, λ*_2_], we employ the bisection method. We test the middle point λ*_m _*= (λ_1 _+ λ_2_)*/*2, and if *r*(λ*_m_*) = *k*, we are done. If *r*(λ*_m_*) *< k*, set λ_2 _= λ*_m_*; otherwise, set λ_1 _= λ*_m_*. Repeat until *r*(λ*_m_*) = *k*.

## Results and discussion

### Application to gene selection and cancer classification

In this section, we apply our proposed AucPR, the penalized logistic regressions, and the AUC based non-parametric method proposed by [3], which maximizes a sigmoid approximation of AUC, to four microarray datasets for gene selection and cancer classification. We refer to our approaches to AucPR with elastic net and lasso penalty as *AucEN *and *AucL*, respectively, the logistic regression approaches with elastic net and lasso penalties as *LogEN *and *LogL*, respectively, and maximizing the sigmoid approximation of AUC as *MSauc *in the following content. The four microarray data sets are:

*· Brain *cancer data: The original data have five different types of tumors, and 42 samples with 5597 expressions. This data set was also studied by [25], and we use their preprocessed data and denote the first two types as the control group and the other three as the case group. It can be downloaded from http://stat.ethz.ch/~dettling/bagboost.html.

*• Colon *cancer data: Expression levels of 40 tumors and 22 normal colon tissues for 2000 human genes, with the highest minimal intensity from 62 subjects measured [26]. The data can be downloaded from *colonCA *package on the Bioconductor website (http://www.bioconductor.org).

*• Leukemia *data: We consider two types of leukemia cancer: acute myeloid leukemia (AML) and acute lymphoblast leukemia (ALL). Samples used by [27] were derived from 47 patients with ALL and 25 patients with AML, with 7129 genes. The data set is available in the *golubEsets *package on the Bioconductor website (http://www.bioconductor.org).

*• DLBCL *data: The diffused large B-cell lymphoma (DLBCL) data set contains 58 DLBCL patients and 19 follicular lymphoma patients from a related germinal center B-cell lymphoma [28]. The data are available from the Broad Institute website (http://www.genome.wi.mit.edu/MPR/lymphoma).

All data sets are further processed using quantile normalization and logarithm transformation (except the *Brain *cancer data, since it has been preprocessed). To save computation time, we screen the genes such that the 1000 genes with the largest absolute moderated t-statistics [29] are kept. Filtering genes by t-typed statistics has been widely used in the literature, for example, [3,5,20] among others. Our empirical study shows that including more than 1000 genes does not significantly change the patterns found. *LogEN *and *LogL *are also implemented by R package *glmnet *and the tuning parameter is chosen by a three-fold CV, using the CV score defined in Equation (4).

**Figure 1:**
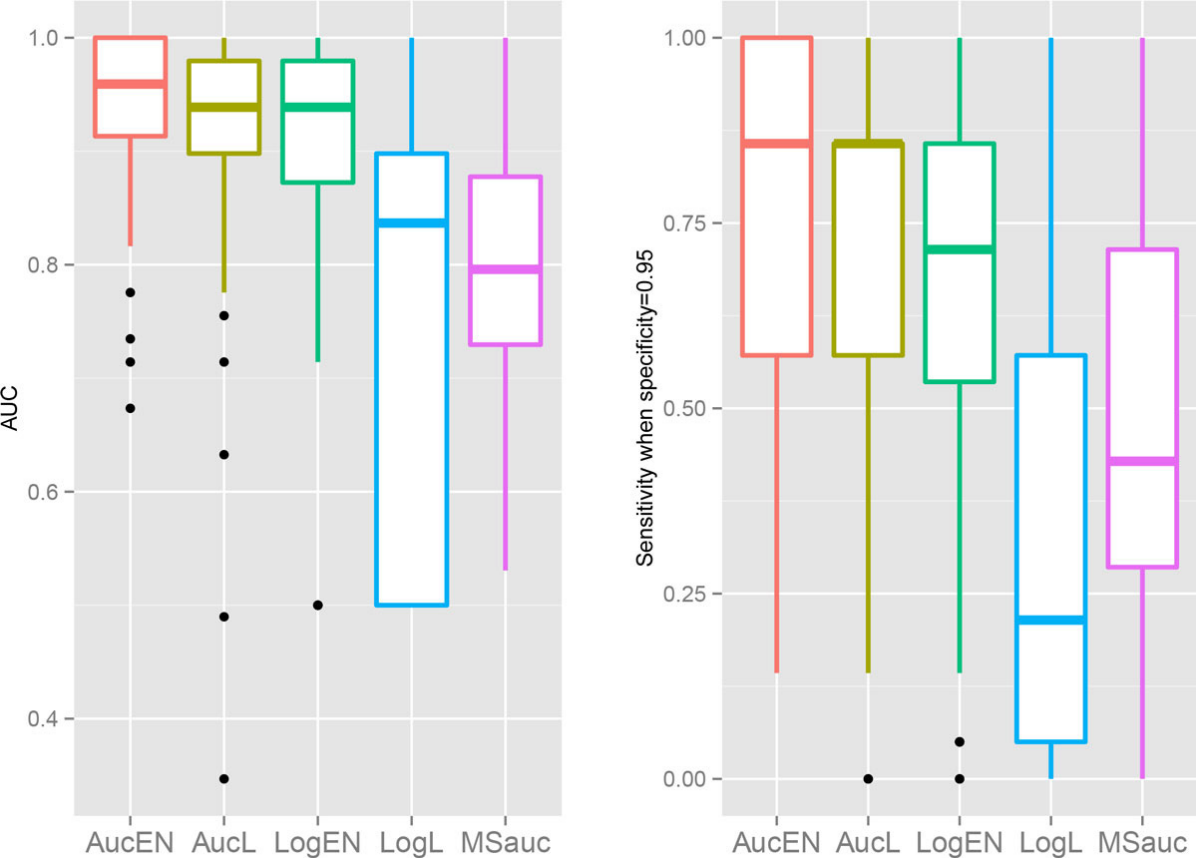
**Box plots in *Brain *cancer data for AUC (left) and sensitivity when specificity equals 0.95 (right)**.

**Figure 2:**
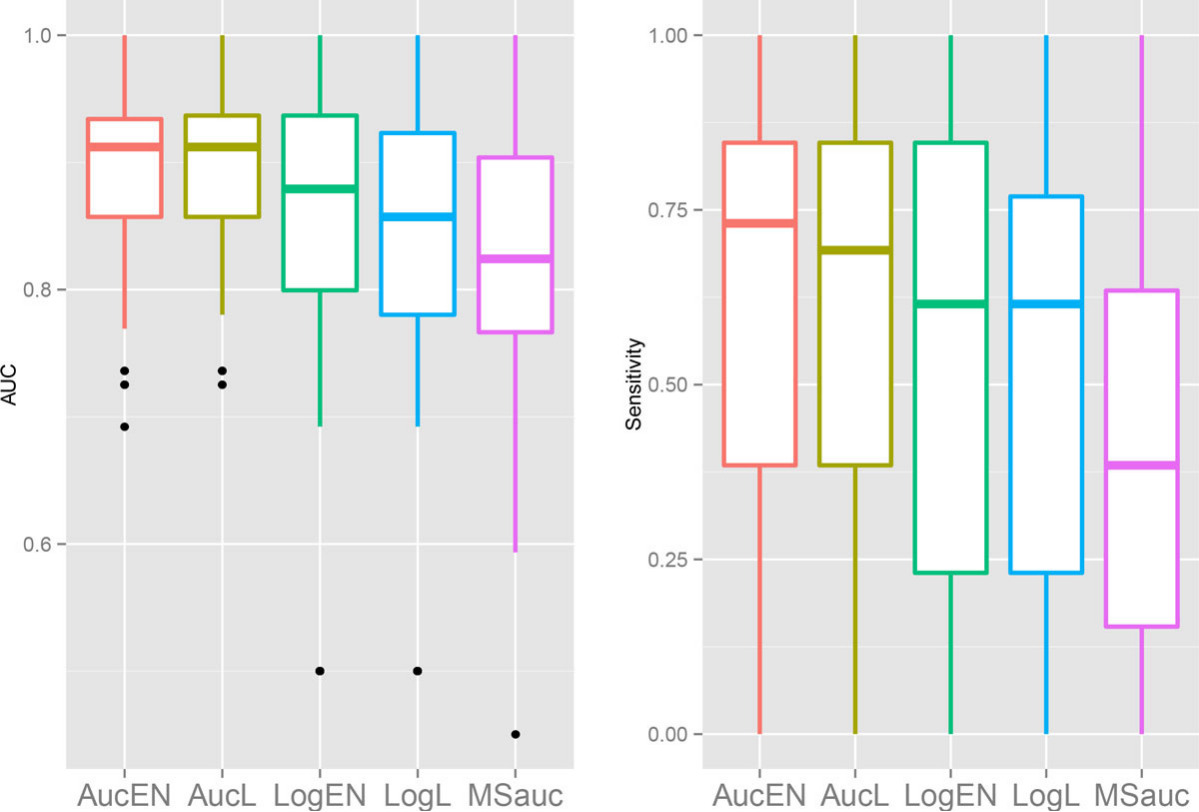
Box plots in *Colon *cancer data for AUC (left) and sensitivity when specificity equals 0.95 (right).

**Figure 3:**
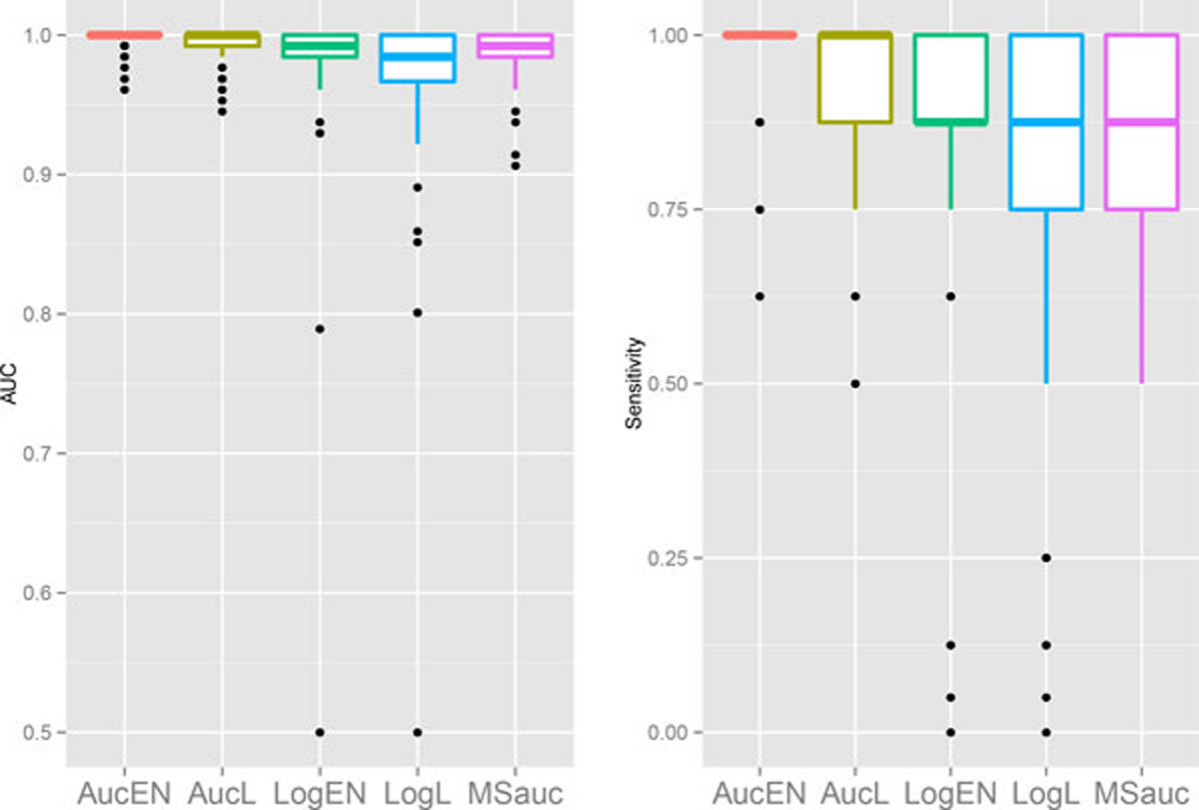
Box plots in *Leukemia *cancer data for AUC (left) and sensitivity when specificity equals 0.95 (right).

**Figure 4:**
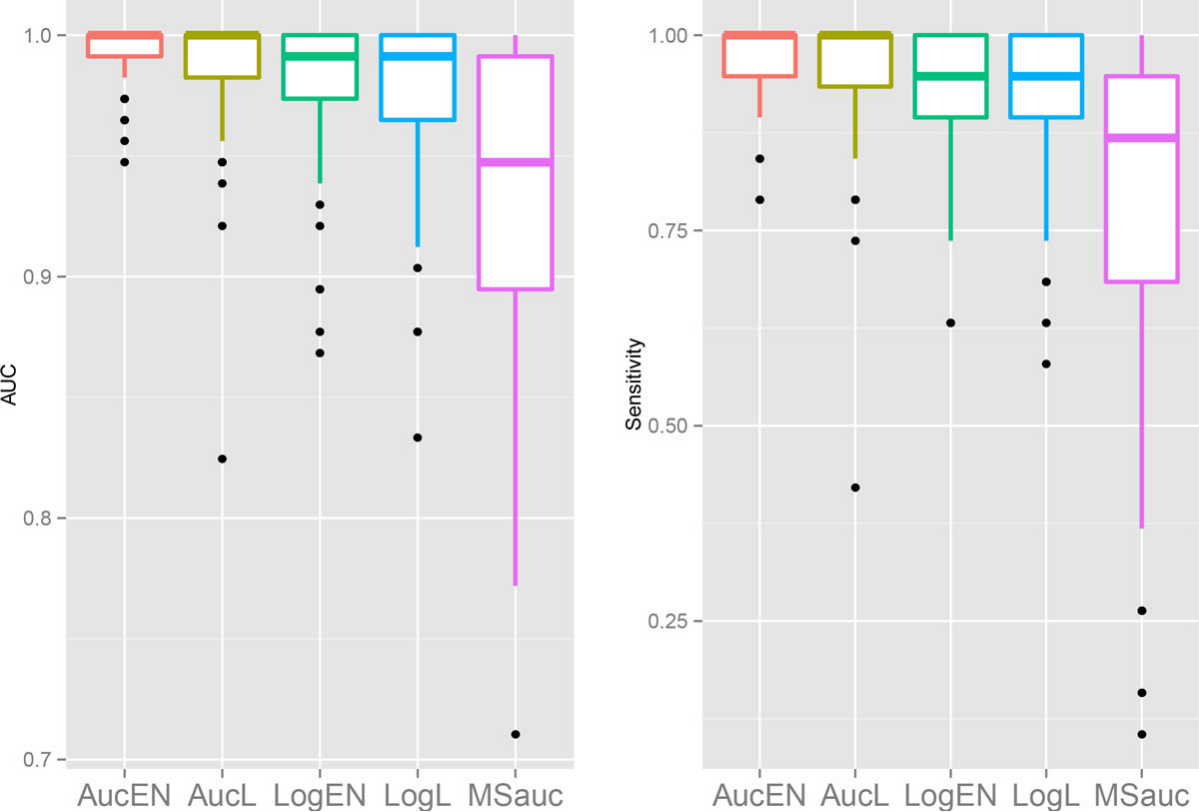
Box plots in *DLBCL *data for AUC (left) and sensitivity when specificity equals 0.95 (right).

Then, we randomly split the data into training and testing sets, comprising 2/3 and 1/3 of the sample, respectively. The AUC value and sensitivity when specificity = 0.95 are evaluated based on the testing set. This procedure is repeated 100 times and the box-plots of the two comparison metrics are plotted in Figures 1 - 4.

We can see that the proposed AucPR outperforms the other approaches for all the four datasets,. The *AucEN *has the best prediction performance. The *AucL *is slightly less powerful than the *AucEN*, but better than the other three methods. The penalized logistic regression and *MSauc *perform poorly for the *Brain *and *Colon *cancer data. Even though the differences of AUC between these approaches are small for *Leukemia *and *DLBCL *data, the superiority of the proposed AUC-based methods becomes larger in sensitivity when specificity is as high as 0.95. This finding is very meaningful, since high sensitivity and high specificity are greatly appreciated for real cancer studies.

**Figure 5:**
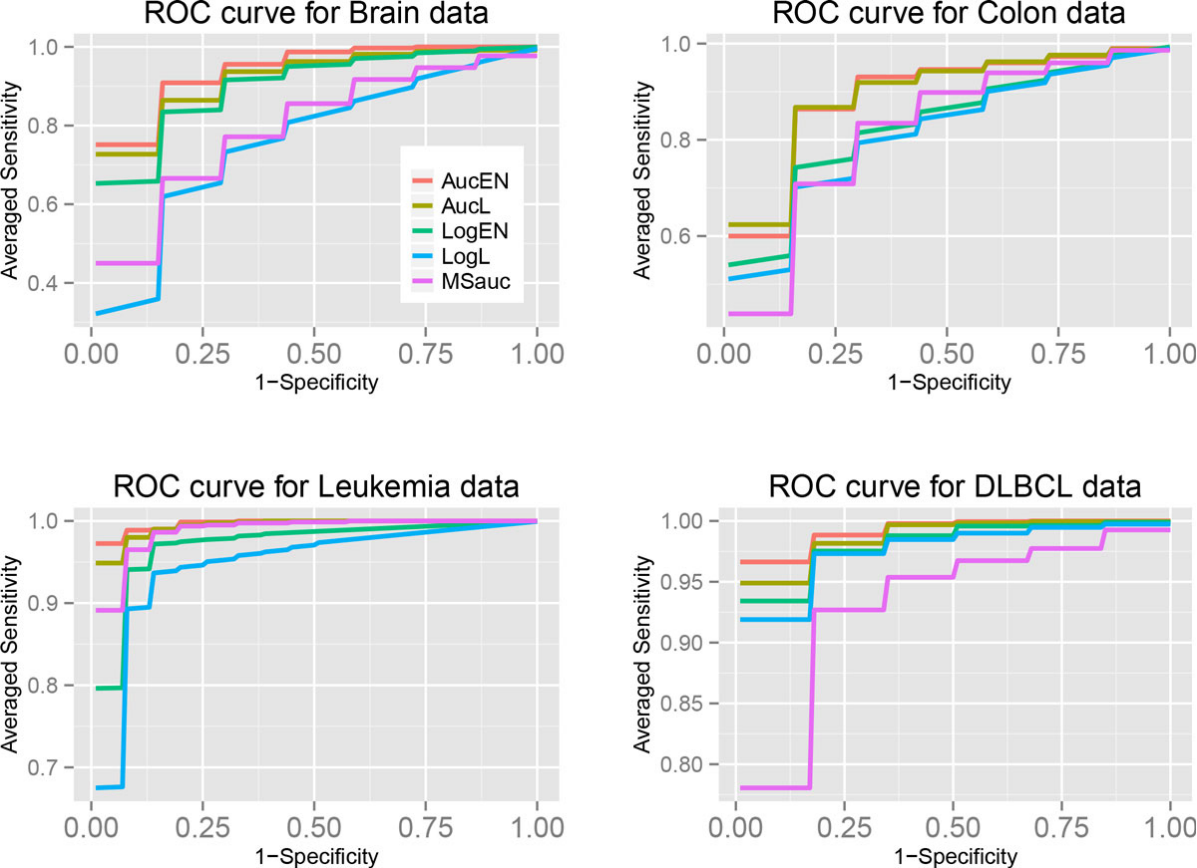
**ROC curves for four real data sets**.

For the four data sets, Table 1 shows the median number of non-zero coefficients for each method. The AucPR selects more markers than the others. The approaches with the elastic net provide more genes than the lasso penalized approaches, which is consistent with the literature [10]. *MSauc *generally selects more genes than the penalized logistic regressions, but does not always give a better prediction performance than the penalized logistic regressions (Figures 1 - 4). The averaged ROC curves are plotted in Figure 5, showing that the ROC curve of the proposed AucPR lies above the curves of others, especially in *Brain *and *Colon *cancer data.

**Table 1:** The median number of genes being selected in four microarray studies.

	*AucEN*	*AucL*	*LogEN*	*LogL*	*MSauc*
*Brain*	42	30	37	3	16
*Colon*	30	22	3	2	22
*Leukemia*	51	36	7	5	10
*DLBCL*	51	35	26	9	17

In summary, the proposed AucPR selects more genes than the other three competing approaches and also have better prediction performance. Although a sparse model is good for interpretation, a better prediction performance is the primary objective and more appealing in many real world applications. As pointed out by [20], sparse models commonly ignore the correlations between the variables, which are generally inefficient even when the zero markers (or "unimportant" markers) are known in advance and all the important markers are selected correctly. It was demonstrated that those unimportant markers are in fact useful and even potentially important for classification because of the correlations. In addition, including a sufficient number of genes to the model has a practical implication; more potential important genes may be incorporated and these genes would have a higher chance for further investigation. In Table 2 the top 10 frequently selected genes by *AucEN *are listed for *Colon, Leukaemia *and *DLBCL *data sets (The gene information is not included in the preprocessed *Brain *cancer data, so we omit those results). The genes which are commonly selected by other approaches are marked. Gene description and related studies in the literature are shown too. The top frequently selected genes by *AucEN *were also reported in the literature.

**Table 2:** Top 10 frequently selected genes by AucEN.

data	gene id	gene symbol	description	Coverage
*Colon*	Hsa.2097	R14852	Human vasoactive intestinal peptide (vip) mrna, complete cds	AucL, LogL, LogEN, [35]
	Hsa.3331	T86473	Nucleoside diphosphate kinase a (Human)	AucL [36]
	Hsa.37937	R87126	Myosin heavy chain, nonmuscle (Gallus gallus)	AucL, LogL, LogEN, Msauc, [3,4]
	Hsa.601	J05032	Human aspartyl-tRNA synthetase alpha-2 subunit mRNA, complete cds	AucL, LogEN, [4]
	Hsa.36952	H43887	Complement factor d precursor (Homo sapiens)	AucL,[37]
	Hsa.8125	T71025	Human (HUMAN)	AucL, [38]
	Hsa.8147	M63391	Human desmin gene, complete cds	AucL, LogL, LogEN, Msauc, [3,4,39]
	Hsa.3306	X12671	Human gene for heterogeneous nuclear ribonucleoprotein (hnRNP) core protein A1	LogL, LogEN, [4]
	Hsa.26673	R76825	RNA-specific gtpase-activating protein (Homo sapiens)	AucL, [40]
	Hsa.14069	T67077	Sodium/potassium-transporting atpase gamma chain (Ovis aries)	[41]

*Leukaemia*	X59711 at	NFYA	NFYA Nuclear transcription factor Y, alpha	[42]
	M30938 at	XRCC5	ATP-DEPENDENT DNA HELICASE II, 86 KD SUBUNIT	[43]
	U57721 at	Kynu	L-kynurenine hydrolase mRNA	[44]
	X07834 at	Sod2	SOD2 Superoxide dismutase 2, mitochondrial	[45]
	U37408 at	Ctbp1	CtBP mRNA	[46]
	M98539 at	ptgds	Prostaglandin D2 synthase gene	[47]
	U35113 at	Mta1	Metastasis-associated mta1 mRNA	[48]
	X13973 at	rnh1	RNH Ribonuclease/angiogenin inhibitor	[49]
	D49817 at	pfkfb3	Fructose 6-phosphate,2-kinase/fructose 2,6-bisphosphatase	[50]
	M83233 at	TCF12	TCF12 Transcription factor 12 (HTF4, helix-loop-helix transcription factors 4)	LogL, LogEN, [51]

*DLBCL*	U96113 at	WWP1	Nedd-4-like ubiquitin-protein ligase WWP1 mRNA, partial cds	AucL, [52]
	U46006 s at	CSRP2	Smooth muscle LIM protein (h-SmLIM) mRNA	AucL, LogL, LogEN, [53]
	M35878 at	igfbp3	INSULIN-LIKE GROWTH FACTOR BINDING PROTEIN 3 PRECURSOR	AucL, [54]
	U77949 at	cdc6	Cdc6-related protein (HsCDC6) mRNA	AucL, [55]
	L41067 at	Nfatc3	Transcription factor NFATx mRNA	AucL, [56]
	U95006 at	STRA13	D9 splice variant A mRNA	[57]
	U64863 at	Pdcd1	HPD-1 (hPD-1) mRNA	AucL, [58]
	AB002409 at	ccl21	SLC	AucL, MSauc, [28]
	HG2279-HT2375 at	TPI1	Triosephosphate Isomerase	AucL
	U17969 at	eif5a	EIF5A Eukaryotic translation initiation factor 5A	[59]

The "Coverage" column shows the genes frequently selected by other methods or reported in the literature.

There are several other popular approaches available for classification in high-dimensional situation. For example, the "SIS" function in package *SIS *(http://cran.r-project.org/web/packages/SIS/index.html), which first implements the Iterative Sure Independence Screening [30], and then fits the final model by penalized regression; tree-based method "randomForest" in randomForest package [31]; *LogEN *with alternative CV score ("type.measure = deviance" in *glmnet *package). As a demonstration, we implemented the third approach on the *Brain *cancer data. The result is improved and has been updated. However, Our method still outperforms others (see Figure 1).

*Remark 2: *Prediction accuracy and interpretation are two major concerns for microarray cancer classification study. A sparse model is generally easier to interpret but may not reflect the true biological phenomena or have poor prediction. For example, many genes are highly correlated in microarray data, and these genes may work together. Therefore, it is worthwhile to identify these genes jointly to increase prediction performance and to provide a sufficient number of potential risks for a further validation study. Note that the lasso penalized logistic regression is too parsimonious, as it cannot select a sufficient number of genes in a highly correlated group and thus, has poor prediction performance, while our *AucL *method, although with the lasso penalty, seems to be able to alleviate this problem by selecting more genes.

### Simulation

In this section, we demonstrate our approaches using synthetic data under two scenarios; genes are generated from a normal distribution or a mixture of normal distributions.

We first simulate gene expressions following a setting similar to [32], where they mimicked the real microarray data. We generate data under a different number of independent blocks (*block *= 1, 2, 3), and the number of genes per block (*size*=5, 20, 40). The data are simulated from multivariate normal distributions *N *(***µ***_*x*_, **Σ**_*x*_(*ρ*)) and *N *(***µ***_*y*_, **Σ**_*y *_(*ρ*)) for diseased and non-diseased classes, respectively. All genes have a variance of 1, and the correlation between genes within a block is *ρ *(*ρ *= 0.3, 0.6, 0.9), whereas the correlation between genes among blocks is 0. In other words, the covariance matrix is a block-diagonal matrix

$$\sum_{x}\left(\rho \right)=\sum_{y}\left(\rho \right)\left(\begin{array}{cccc} A\left(\rho \right) & 0 & \cdots \; & 0\\ - & A\left(\rho \right) & \cdots \; & 0\\ \vdots & \vdots & \vdots & \vdots \\ 0 & 0 & 0 & A\left(\rho \right) \end{array}\right),$$

where

$$A\left(\rho \right)=\left(\begin{array}{cccc} 1 & \rho & \cdots \; & \rho \\ \rho & 1 & \cdots \; & \rho \\ \vdots & \vdots & \vdots & \vdots \\ \rho & \rho & \cdots \; & 1 \end{array}\right)$$

The mean vectors are set as ***µ****_y _*= (0.6, 0.6*,... *, 0.6) and ***µ****_x _*= (−0.6*, −*0.6*, ..., −*0.6). Here the mean vectors are selected such that the AUC of each single gene is 0.8.

After the informative genes described above are generated, we evenly add a type of non-informative "genes" from *N *(0, 1) and another type of non-informative "genes" from *U *[−1, 1], for both diseased and non-diseased observations, and make 1000 markers in total.

We generate *n *= *m *= 40 i.i.d. individuals as a training set for diseased and non-diseased samples, respectively, from the above distributions. Under the same structure as the training set, another *n *= *m = *20 samples are simulated independently as a testing set. Each method is applied to the training set and the prediction performance is measured on the testing set. We repeat this procedure 100 times, as we have done in the examples with real data.

For the synthetic data, the AucPR shows better prediction accuracy than the other three approaches in most scenarios. The median values of AUC, sensitivity when specificity = 0.95, the number of true informative markers being selected (*nIMS *), and the number of total markers being selected (*nTMS *), are summarized in Tables 3, 4, 5. There are some facts we can state, based on the simulation results:

**Table 3:** Summary of simulation results for different sizes of each block, when *ρ *= 0.6 and *block *= 1 under a normal scenario. *nIMS *and *nTMS *stand for the number of the true informative markers selected and the total number of markers selected, respectively.

*Size*	*Method*	*Auc*	*Sensitivity*	*nIMS*	*nTMS*
5	*AucEN*	0.84	0.50	3	4
	*AucL*	0.82	0.45	3	4
	*LogEN*	0.82	0.45	2	2
	*LogL*	0.80	0.40	1	1
	*MSauc*	0.81	0.40	1	4

40	*AucEN*	0.86	0.55	20	24
	*AucL*	0.86	0.55	13	18
	*LogEN*	0.85	0.50	4	4
	*LogL*	0.82	0.45	2	2
	*MSauc*	0.81	0.45	1	3

**Table 4:** Summary of simulation results for different *ρ*, when *size *= 5 and *block *= 1, under a normal scenario.

*ρ*	*Method*	*Auc*	*Sensitivity*	*nIMS*	*nTMS*
0.3	*AucEN*	0.81	0.45	3	12
	*AucL*	0.81	0.45	3	6
	*LogEN*	0.85	0.50	3	3
	*LogL*	0.85	0.50	2	2
	*MSauc*	0.82	0.45	2	6

0.9	*AucEN*	0.81	0.45	3	4
	*AucL*	0.81	0.45	2	2
	*LogEN*	0.80	0.40	2	2
	*LogL*	0.80	0.40	1	1
	*MSauc*	0.79	0.40	1	3

**Table 5:** Summary of simulation results for different *block*, when *size *= 20 and *ρ *= 0.6, under a normal scenario.

*block*	*Method*	*Auc*	*Sensitivity*	*nIMS*	*nTMS*
1	*AucEN*	0.86	0.55	12	14
	*AucL*	0.85	0.55	10	12
	*LogEN*	0.83	0.47	4	4
	*LogL*	0.81	0.40	2	2
	*MSauc*	0.81	0.40	1	4

3	*AucEN*	0.96	0.85	25	40
	*AucL*	0.95	0.85	20	32
	*LogEN*	0.95	0.85	16	16
	*LogL*	0.94	0.75	8	8
	*MSauc*	0.92	0.70	10	31

1 Given *ρ *and the number of blocks (*block*), as the block *size *increases, our AucPR dominates the other approaches. We summarize the results when *size *= 5 and *size *= 40 in Table 3.

2 Given block *size *and the number of blocks, as *ρ *becomes larger, the performance of our methods do not vary much, while those of the other three methods become worse. Specifically, the sensitivities of our methods are getting larger than others when *ρ *is getting larger. The results for *ρ *= 0.3 and *ρ *= 0.9 are given in Table 4.

3 As the number of independent blocks increases, all methods have improved performances. When the number of blocks is 3, except *LogL *and *MSauc*, the other three methods seem to be similar in each case with *AucEN *performing slightly better (Table 5).

4 Penalized logistic regression performs better only when *ρ *is small (for example, 0.3) and the number of the informative genes is small. Approaches with elastic net penalty always lead to better results than the approaches with lasso penalty (Tables 3, 4, 5).

5 Generally, our AucPR approaches select more informative genes, and the approaches with elastic net penalty incorporate more informative genes than the approaches with lasso penalty (Tables 3, 4, 5). Note that as *block *and/or *size *increase (or equivalently, as the number of informative genes increases), the number of selected informative genes for our AUC-based methods increase faster, but logistics regression based approaches and *MSauc *do not. This fact may be interpreted as that our approaches show better prediction accuracy.

Next, we also study the scenario where the genes are generated from a non-Gaussian setting. We simulate 50 informative genes from 0.8*N *(***µ***_*y*_, **Σ**_*y *_(0.8)) + 0.2*N *(**0*, I***) and 0.8*N *(***µ***_*x*_, **Σ**_*x*_(0.8))+0.2*N *(**0*, I***) for diseased and non-diseased groups, respectively. The non-informative genes are generated in the same way as in the first scenario. Similar patterns can be found as in the normal distribution scenario (data are not shown).

In summary, through selecting more genes, the proposed AucPR performs better when there are a lot of informative genes or the correlations between them are high (larger than 0.6 for example).

**Figure 6:**
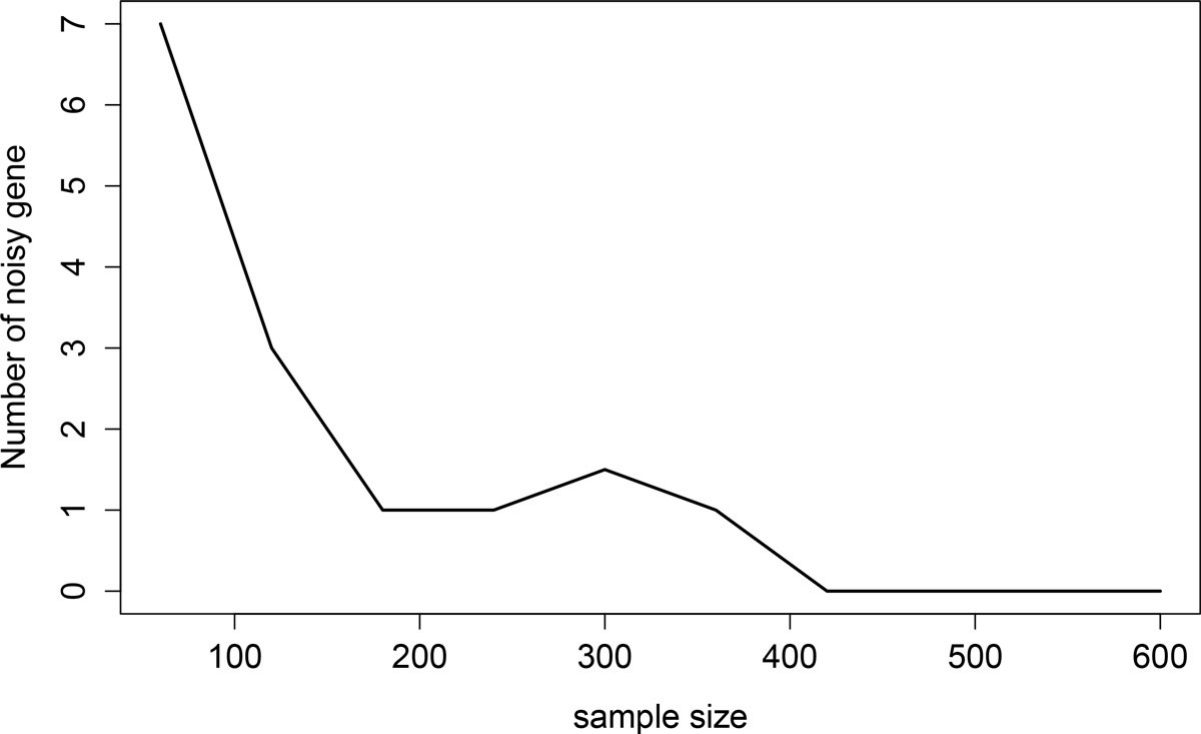
The number of noisy genes selected by *AucL *vs. the sample size of the simulation study, with *ρ *= 0.6, *block *= 2, and *size *= 20.

*Remark 3: *Note that the penalized logistic regressions are very powerful for marker selection in the sense that all the selected genes are the true informative genes, that is, *nIMS *= *nTMS*. For AucPR, the *nTMS *is larger than the *nIMS*, that is, there are some noisy genes selected. If the sample size increases, this phenomenon can be avoided or become negligible. Figure 6 shows that when the sample size is larger than 100, the number of noisy genes selected by *AucL *becomes very small.

### Discussion

Note that, in our comparison study, the tuning parameters for all methods are tuned with an empirical (non-parametric) AUC estimator as the CV score. When sample size is very small, some difficulties may occur for calculating such AUC estimators as we did in the *brain *cancer study. Alternatively, parametric AUC estimators or the deviance from a distribution model can be used as the CV score. Different CV scores may lead to different results, especially when the sample sizes are small. It is worthy of investigating this issue as a future research topic.

Although we only use gene expression microarray data, AucPR can also be applied to other types of high-throughput omics data, such as miRNA and protein data.

AucPR methods rely on sample mean vectors and sample covariance matrices, which may not be stable enough, specifically when only a small number of samples are available. An improvement may exist in practice by replacing them with, for example, sample median and the positive-definite estimator of a large covariance matrix proposed by [33]. This can be a topic of future research.

Note that after the transformation, we try to solve a regression problem with p "samples" and p "predictors." Thus, the computation cost would grow quickly as p increases. Although screening the original p genes to a smaller number (1000 in our numerical studies) of genes is widely used and does not affect the prediction performance, as seen from our empirical study and the relevant literature [3,5,13,20], it is still worthwhile to develop fast algorithms for large scale and high-dimensional regression problem. This, too, needs further investigation.

## Conclusions

We propose a powerful parametric and easily-implementable linear classifier AucPR, for gene selection and disease prediction for high-dimensional data. We transform a classical parametric AUC estimator into a linear regression and thus, the existing packages for regularized linear regression can be used directly. This novelty makes the implementation of the proposed methods very easy and efficient, since the regularized regression has been well studied. The proposed parametric method also avoids maximizing a non-concave objective function and elaborately choosing the smoothing parameter in a conventional non-parametric method. Comparisons among the AucPR, the penalized logistic regression, and a non-parametric AUC-based approach shows that our methods lead to better classifiers in the sense of predictive performance, through application to real microarray and synthetic data. In addition, the proposed AucPR selects more markers than the others and thus, could include more potential important markers for further investigation.

In addition, [34] demonstrated that the linear combination of multiple markers based on maximizing AUC generally performs better than logistic regression when the logistic model does not hold, and the two methods are comparable when the logistic model is satisfied, but their analysis was done under the condition that a very limited number of markers would be considered. This paper states that the AUC-based approach could also be advocated in high-dimensional setting, since it achieves better prediction ability than the penalized logistic regression.

## Availability and supporting data

This work was implemented in R software. The R source codes are freely available at http://bibs.snu.ac.kr/software/aucpr.

## Competing interests

The authors declare that they have no competing interests.

## Authors' contributions

WY and TP designed the method. WY performed the analyses. WY and TP interpreted the results and wrote the manuscript.

## Declarations

Publication charges for this work was funded by the National Research Foundation of Korea(NRF) grants funded by the Korea government(MSIP) (No. 2012R1A3A2026438 and 2008-0062618).

This article has been published as part of *BMC Genomics *Volume 15 Supplement 10, 2014: Proceedings of the 25th International Conference on Genome Informatics (GIW/ISCB-Asia): Genomics. The full contents of the supplement are available online at http://www.biomedcentral.com/bmcgenomics/supplements/15/S10.
